# Protective Effect of Food Against Inactivation of Human Coronavirus OC43 by Gastrointestinal Fluids

**DOI:** 10.1007/s12560-022-09520-5

**Published:** 2022-03-23

**Authors:** Jennifer Harlow, Matthew Dallner, Neda Nasheri

**Affiliations:** 1 National Food Virology Reference Centre, Bureau of Microbial Hazards, Health Canada, Ottawa, ON Canada; 2 Department of Biochemistry, Microbiology and Immunology, Faculty of Medicine, University of Ottawa, Ottawa, ON Canada

**Keywords:** Human coronavirus OC43, Gastrointestinal fluids, Survival, Infectivity assay

## Abstract

The involvement of the gastrointestinal (GI) tract in severe acute respiratory syndrome coronavirus-2 (SARS-CoV-2) infection has been reported in multiple studies. Since it has been demonstrated that human intestinal epithelial cells support productive viral replication and that a substantial portion of infected individuals shed the virus in feces, the possibility of fecal–oral and fecal-respiratory modes of transmission have been proposed for SARS-CoV-2. In order to establish viral replication in the intestine, enteric viruses need to retain their infectivity in often low pH gastric fluids, and in intestinal fluids, which contain digestive enzymes and bile salts. In this study, we examined whether human coronaviruses OC43 (HCoV-OC43) can remain infectious in simulated GI fluids that models human fasting-state and fed-state, in the presence or absence of food. We demonstrated that except for fasting-state gastric fluid (pH 1.6), the virus can remain infectious in all other gastrointestinal fluids for 1 h. Furthermore, we demonstrated that presence of food could significantly improve viral survival in gastric fluids. Therefore, this study provides evidence that ingestion with food could protect the virus against inactivation by the GI fluids.

**Supplementary Information:**

The online version contains supplementary material available at 10.1007/s12560-022-09520-5.

## Introduction

Coronaviruses are enveloped viruses with a genome composed of a non‐segmented positive sense single-stranded RNA with a size of approximately 30 kb (Fehr & Perlman, 2015). Based on phylogenetic clustering, coronaviruses are divided into four genera: Alphacoronavirus, Betacoronavirus, Gammacoronavirus and Deltacoronavirus. Coronaviruses that infect humans (HCoVs), belong to the alpha and beta genera. Severe acute respiratory syndrome (SARS)-CoV-2, which is responsible for the coronavirus disease 2019 (COVID-19) pandemic, is a Betacoronavirus that causes mild to severe respiratory infections, as well as gastrointestinal symptoms (Cimolai, 2020; Kariyawasam et al., 2021). Human-to-human transmission of SARS-CoV-2 is mainly attributed to contaminated respiratory droplets and aerosols (Meyerowitz et al., 2021). Although gastrointestinal (GI) manifestations are reported in 11.4–61.1% of COVID-19 patients (Kariyawasam et al., 2021; Leal et al., 2021) and multiple lines of evidence suggest that the infectious viral particles are excreted in the feces of 41% of infected individuals (Britton et al., 2021; Shi et al., 2020), there is no conclusive evidence for fecal–oral or fecal-respiratory transmission.

It has been reported that the viral load in feces could reach to 10^7^ RNA copies/g, even higher than that in nasopharyngeal swabs (Wolfel et al., 2020). The presence and persistence of such large amounts of viral RNA in feces cannot only be explained by ingestion of viral particles replicated in the respiratory system, but suggests potential replication of SARS-CoV-2 in the intestinal tract (Guo et al., 2021). Angiotensin-converting enzyme 2 (ACE2), which is the main receptor for SARS-CoV-2 entry, is abundantly expressed in the lung and upper respiratory epithelia, as well as in the duodenum and small intestine, with lower levels in the stomach and large intestine (Hikmet et al., 2020). Although the mere expression of viral receptors in the GI system does not mean these cells are permissive to respiratory virus infection. The possibility of enteric propagation is further supported by the fact that human intestinal cells are highly permissive to infection with SARS-CoV- 2 (Lamers et al., 2020; Zhou et al., 2020). The ability to retain infectivity in the GI fluids is essential for a microbe to establish infection in the human intestinal tract, and it has been reported that SARS-CoV, SARS-CoV-2, middle-east respiratory syndrome (MERS), and HCoV-229E can tolerate fed-state gastric fluid and fasting-state intestinal fluid, but not fasting-state gastric fluids and fed-state intestinal fluids (Chak-Yiu Lee et al., 2020; Zhou et al., 2017).

Coronaviruses are enveloped, and as such, are expected to be susceptible to gastric acid and bile, therefore theoretically, it is unlikely that these viruses retain their infectivity and reach the lower GI tract to be excreted in the feces (Hirose et al., 2017). On the other hand, many of the mammal and avian‐associated coronaviruses are well known to cause gastroenteritis in their host species, including poultry, swine, bovine, equine, canine, and feline hosts (Cimolai et al., 2020; Pusterla et al., 2018; Wang, et al., 2019)**.** Furthermore, evidence of the presence of SARS-CoV has been found in the intestinal mucosal epithelium and lymphoid tissue of human fatal cases (Gu et al., 2005; Shi et al., 2005). Notably, SARS-CoV-2 antigen has been detected in duodenum of infected golden hamster model (Sia et al., 2020). These observations suggest that at least some coronaviruses are resistant to gastrointestinal fluids and enzymes.

Given the limited access to biosafety level 3 (BSL-3) containment facilities required to safely handle SARS-CoV-2, scientists have turned to surrogate viruses that enable studies of viral replication as well as survival and inactivation at biosafety level 2 (BSL-2). This approach has expanded the current knowledge regarding the molecular and applied aspects of highly pathogenic coronaviruses.

The aim of this study is to examine the potential protective effect of food against HCoV inactivation by gastrointestinal fluids, using HCoV-OC43 as a surrogate for more pathogenic coronaviruses. In a previous study, we demonstrated that HCoV-229E and HCoV-OC43 survive significantly longer on cucumbers than apples and tomatoes (96 h vs 16 h) when stored at 22 °C (Blondin-Brosseau et al., 2021). As an extension of the previous study, we investigated the potential for HCoV to remain infectious following ingestion, in the presence or absence of cucumber slices.

## Materials and Methods

### Cell line and Virus

Human lung fibroblast cells, MRC-5 (ATCC# CCL-171), and human coronavirus OC43 (HCoV-OC43; ATCC# VR-1558) were obtained from the American Type Culture Collection (ATCC) (Cedarlane, Canada). Cells were grown and maintained at 37 °C and 5% CO_2_ in culture media composed of Eagle’s minimal essential medium, supplemented with 0.22% (w/v) sodium bicarbonate, 500 µg/mL penicillin/streptomycin), 1% Glutamax-1, 1% non-essential amino acids, 1% amphotericin B and 10% (v/v) fetal bovine serum (FBS) (ThermoFisher Scientific Inc, Canada). MEM maintenance media was used for dilution media of experimental samples, with the same composition as growth media but with 2% FBS.

### ***Quantification of HCoV-OC43 Using Median Tissue Culture Infectious Dose (TCID***_***50***_***) Assay***

Quantification of infectious HCoV-OC43 was conducted as described previously (Dallner et al., 2021). Briefly, MRC-5 cells were grown for 2 to 3 days and were seeded in 96 well plates at a cell concentration of 5 × 10^4^ cells/100 µL well, up to 24 h in advance of the assay, in order to reach 90% confluence. Maintenance media was used to dilute gastric fluid treated, intestinal fluid treated, or maintenance media control samples. Cell monolayers were washed once with 1 × PBS (pH 7.35) and 100 µL of each dilution was used for quantification of tissue culture infectious dose, measured in quadruplicate for each sample. HCoV-OC43 stock was used as the positive control, diluted in maintenance media. Uninoculated maintenance media was the negative control. MRC-5 plates were then incubated at 33 °C for 5 days. Samples were removed from the 96 well plate, discarded, and stained with 0.1% crystal violet. Cells were observed under the microscope and the cytopathic effect for each sample was recorded. TCID_50_/mL was calculated using the Reed-Muench method (Reed & Muench, 1938). TCID_50_ values were converted to PFU by multiplying by 0.7, which is a constant value obtained based upon Poisson distribution (ATCC, 2021). The percentage viral recovery of HCoV-OC43 from 2 g cucumbers was determined by this equation:

$$Viral \,recovery \left(\%\right)=\frac{Recovered\, infectious \, virus (PFU)}{Inoculated \, infectious\, virus\, (PFU)}\times 100$$

### Sample Preparation and HCoV-OC43 Treatment with Simulated Gastrointestinal Fluids

English cucumbers (PLU code 4593, Ontario, Canada) were purchased from a local grocery store in Ottawa, Ontario, Canada. Simulated gastric and intestinal fluids were prepared according to manufacturer’s instructions (Biorelevant, London, UK). Simulated gastric fluids were supplemented with 0.1 mg/mL pepsin (Millipore Sigma, Canada). The surface of intact cucumbers were disinfected with 70% ethanol, dried, and chopped into 2 g amounts. Each 2 g sample of cucumber was inoculated, in triplicate, with 100 µL of 10^6^ TCID_50_/mL HCoV-OC43, applied as drops to provide an even cover of the cucumber surface, then left at room temperature for 10 min to dry. Each sample was then mixed with 5 mL of fed-state gastric fluid, (FEDGAS pH 6, pH 4.5, or pH 3), fasting-state gastric fluid (FaSSGF, pH 1.6), fed-state intestinal fluid (FeSSIF pH 5), or fasting-state intestinal fluid (FaSSIF pH 6.5). Instead of gastric or intestinal fluid, maintenance media was added to inoculated cucumber samples as a control and to calculate viral recovery. Samples were then incubated at 37 °C for 0, 10 or 60 min with rocking at 320 rpm. At the end of incubation, the gastric or intestinal fluid was neutralized to pH 7 using 2.5 M sodium hydroxide, and then processed immediately for viral quantification.

### HCoV-OC43 Treatment with Simulated Gastrointestinal Fluids Without Food

Simulated gastric and intestinal fluids were prepared as described above. In triplicate, 100uL of 10^6^ TCID_50_/mL HCoV-OC43 was added directly to each type of gastric or intestinal fluid and incubated at 37 °C for 0, 10 or 60 min with rocking at 320 rpm. The gastric or intestinal fluid was neutralized to pH 7 using 2.5 M sodium hydroxide and samples were processed immediately.

### Statistical Analysis

Statistical analysis was performed using GraphPad Prism v9.0 (GraphPad Software, San Diego, CA) (2-way ANOVA multiple comparisons) was used to determine significant differences between treatments. Student t-test was used to determine the statistical significance of the difference between treatments in the presence or absence of cucumber.

## Results and Discussion

In order to determine whether HCoV-OC43 can survive in gastrointestinal fluid, 7 × 10^4^ PFU/mL (1 × 10^5^ TCID_50_/mL) of the virus, in the presence or absence of food (cucumber slices), were exposed to fasting (pH 1.6) and fed-state gastric fluids (pH 3, 4.5, and 6), supplemented with pepsin, as well as fasting- and fed-state intestinal fluids, pH 6.5 and 5, respectively, for 0 to 60 min. The extraction efficiency of HCoV-OC43 from cucumber slices in the presence of maintenance media was 11.1%. The detection limit (LOD) for HCoV-OC43 on cucumbers was previously determined by our group to be 32 PFU (Dallner et al., 2021).

**Fig. 1:**
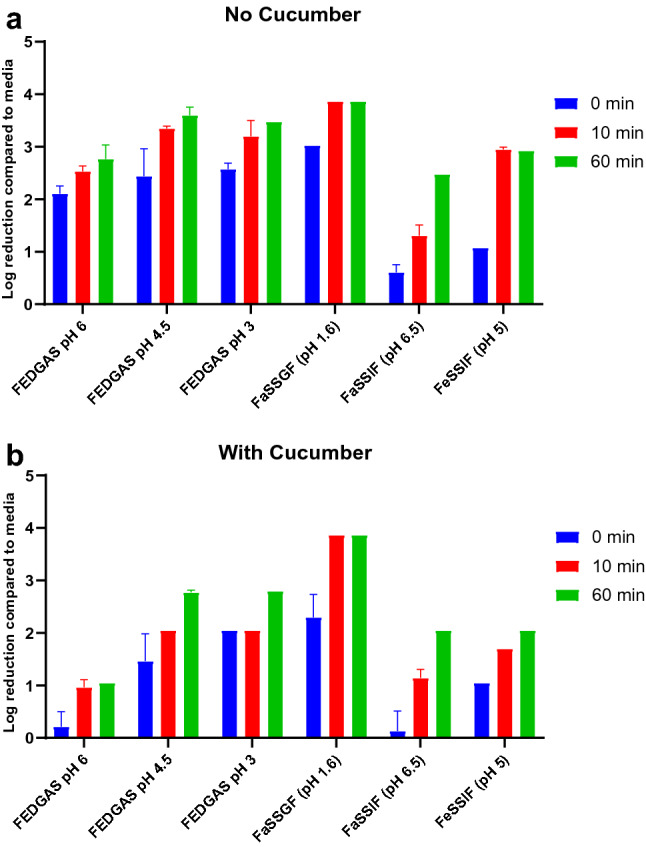
In vitro stability of HCoV-OC43 in simulated human gastrointestinal fluids **a** in the absence of food **b** on two grams of cucumber slices. HCoV-OC43 inoculum (10^5^ TCID_50_) was added directly to each type of gastric or intestinal fluid and incubated at 37 °C for 0, 10 or 60 min. FaSSIF: fasting-state simulated intestinal fluid. FeSSIF: fed-state simulated intestinal fluid. FEDGAS: fed-state gastric fluid. FaSSGF: fasting-state gastric fluid. Data are presented as log reduction in viral infectivity compared to the media treatment. Data obtained from three independent experiments. Error bars are standard deviations

Figure 1a demonstrates the log reduction compared with the media control following treatments in the absence of food and Fig. 1b demonstrates the log reduction in viral infectivity in the presence of food. As shown in Fig. 1a, and b, HCoV-OC43 infectivity was rapidly lost after 10 min of treatment with the fasting-state gastric fluid (FaSSGF, pH 1.6). The presence of food did not lead to recovery of any infectious virus after treatment with (FaSSGF, pH 1.6) for 10 and 60 min (Fig. 1b, and Supplementary Table 1). We compared this to fed-state gastric fluids (FEDGAS) at different stages of stomach emptying (early: pH 6, mid: pH 4.5, and late: pH 3), as well as fed-state intestinal fluid (FeSSIF, pH 5) and fasting-state intestinal fluid (FaSSIF, pH 6.5). The results showed that HCoV-OC43 remain infectious for 60 min in all other types of fed-state gastric fluids and both intestinal fluids. However, the infectivity was reduced in fluids modeling the mid and late stages of stomach emptying (FEDGAS pH 4.5 and pH 3, respectively) compared to FEDGAS pH 6.5, possibly due to the higher acidic conditions. Importantly, the presence of food significantly improved the viral survival in FEDGAS pH 6.5, 4.5 and pH 3 (Supplementary Table 1). However, the presence of food did not lead to a significant difference in viral survival in the presence of fed-state or fasting-state intestinal fluids (Supplementary Table 1). In general, HCoV-OC43 retained the most infectivity at the fasting-state intestinal fluids, followed by the early stage fed-state gastric fluid (pH 6).

Treatment with FaSSGF (pH 1.6) rapidly inactivated the virus, at T_0_ the infectious viral titer was reduced by more than 2 log and longer treatment times of 10 and 60 min completely inactivated the virus (< LOD, i.e. 3.8 log reduction in infectivity). This finding is in accordance with previous studies that have reported complete inactivation of HCoVs in fasting-state gastric fluids (Chak-Yiu Lee et al., 2020; Zhou et al., 2017). Consistent with previous reports, treatment with each of the GI fluids at 10 and 60 min significantly reduced viral infectivity compared to the treatment with maintenance media. Furthermore, there was a significant decrease in viral infectivity following treatment with FeSSIF, compared to FaSSIF at T_0_. Potentially, this was due to the deleterious effect of bile on the viral membrane. However, in contrast to previous studies (Chak-Yiu Lee et al., 2020; Zhou et al., 2017), treatment with FeSSIF in the presence or absence of cucumber, did not lead to complete inactivation of HCoV-OC43 (Fig. 1a, and b). Nonetheless, in the absence of food, there is approximately 3 logs reduction in viral infectivity at 10 and 60 min treatment with FeSSIF (Supplementary Table 1).

Our results are in accordance with the observations made in a study from COVID-19 patients undergoing gastrointestinal endoscopy, in which SARS-CoV-2 RNA was detected and quantified in the gastric fluid and intestinal fluids obtained (Miyake et al., 2021). This indirectly indicates that the virus was able to endure gastrointestinal fluids.

While fecal-respiratory and fecal–oral modes of transmission have been demonstrated for some respiratory viruses including Hantavirus and Nipah virus (O'Brien et al., 2020; Witkowski et al., 2017), these modes of transmission remain controversial for SARS-CoV-2 (Britton et al., 2021). To date, only a few reports have indicated the possibility of fecal-respiratory transmission of SARS-CoV-2 (Meyerowitz et al., 2021). For example, one study demonstrated that exposure to a ruptured sewage pipe might have led to an outbreak of COVID-19 in a passenger ship (Colson et al., 2021) where travelers and crewmembers were infected with the same virus without being in direct contact with each other (Dergham et al., 2021; Guo et al., 2021). In the context of the COVID-19 pandemic, it is still unclear how gastrointestinal virus replication might affect the clinical outcome of infection and the transmission dynamics in the population. Further studies are required to determine whether SARS-CoV-2 shed in fecal matter can transmit COVID-19 between susceptible hosts. Addressing this knowledge gap will allow for understanding the potential role of fecal–oral and fecal-respiratory routes in COVID-19 transmission, which in turn will aid in the implementation of effective disease control measures.

Altogether, this study demonstrates that coronaviruses could be remain infectious in human GI fluid if ingested, and therefore, it is possible that infection could begin in the intestine. Nevertheless, it seems that the proportion of the virus that remains infectious in stool is not remarkable (Cuicchi et al., 2021).

## Supplementary Information

Below is the link to the electronic supplementary material.

Supplementary file1 (DOCX 18 kb)

## Data Availability

The information is provided in the article. Further details can be provided upon request.
