# Supplementary material for: Protective Effect of Food Against Inactivation of Human Coronavirus OC43 by Gastrointestinal Fluids
Source: Food Environ Virol. 2022 Mar 23;14(2):212–6. doi: 10.1007/s12560-022-09520-5 (PMC8941299; doi:10.1007/s12560-022-09520-5)
Supplement: Supplementary file 1 — Supplementary file1 (DOCX 18 kb) [file 12560_2022_9520_MOESM1_ESM.docx]

**Supplementary material**

**Supplementary Table 1.** Comparison of HCoV-OC43 inactivation upon treatment with simulated GI fluid in the presence or absence of food. Average log reduction compared with treatment to media. Data obtained from three independent experiments. NF: no food, WF: with food. FaSSIF: fasting-state simulated intestinal fluid. FeSSIF: fed-state simulated intestinal fluid. FEDGAS: fed-state gastric fluid. FaSSGF: fasting-state gastric fluid. Statistical analysis was performed using student t-test. * represents p-value < 0.05.

|  | **0 min** | | **10 min** | | **60 min** | |
| --- | --- | --- | --- | --- | --- | --- |
|  | **NF** | **WF** | **NF** | **WF** | **NF** | **WF** |
| **FEDGAS pH 6** | 2.11* | 0.21 | 2.53* | 0.96 | 2.77* | 1.05 |
| **FEDGAS pH 4.5** | 2.44* | 1.46 | 3.35* | 2.05 | 3.6* | 2.77 |
| **FEDGAS pH 3** | 2.57* | 2.05 | 3.2* | 2.05 | 3.47* | 2.8 |
| **FaSSGF (pH 1.6)** | 3.02 | 2.3 | 3.86 | 3.86 | 3.86 | 3.86 |
| **FaSSIF (pH 6.5)** | 0.61 | 0.13 | 1.31 | 1.14 | 2.47 | 2.05 |
| **FeSSIF (pH 5)** | 1.07 | 1.05 | 2.95 | 1.7 | 2.92 | 2.05 |
